# Correction to: Expanding the potential genes of inborn errors of immunity through protein interactions

**DOI:** 10.1186/s12864-021-08013-2

**Published:** 2021-10-15

**Authors:** Humza A. Khan, Manish J. Butte

**Affiliations:** 1 Division of Immunology, Allergy, and Rheumatology, Department of Pediatrics, University of California Los Angeles, 10833 Le Conte Ave, MDCC Building, Room 12-430, Los Angeles, CA 90095 USA; 2 Department of Microbiology, Immunology, and Molecular Genetics, University of California Los Angeles, 10833 Le Conte Ave, MDCC Building, Room 12-430, Los Angeles, CA 90095 USA

**Correction to:** ***BMC Genomics*** **22, 618 (2021)**

**https://doi.org/10.1186/s12864-021-07909-3**

Following publication of the original article [1], it was reported that due to a typesetting error that the figure labels in the horizontal axes overlaid a previous version in Fig. 3 and that the Additional files 1-8 were incorrectly uploaded in CSV format instead of XSLX.

**Fig. 3:**
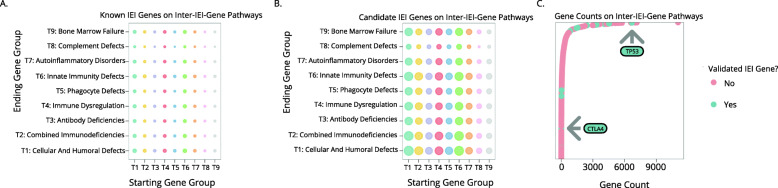
Discovery Pathway 2 uses protein interaction data to describe pathways between genes. **A** Bubble plot showing the relative numbers of known IEI genes that lie on protein interaction pathways between known IEI genes and known IEI genes. **B** Bubble plot showing the relative numbers of candidate IEI genes that lie on protein interaction pathways between known IEI genes and known IEI genes. **C** Number of times that a particular gene appears along different pathways between known IEI genes and known IEI genes. Two previously known IEI-causative genes are indicated

The correct versions of Fig. 3 and the Additional files are included in this Correction article and the original article has been corrected.

## Supplementary Information

**Additional file 1: Table S1.** IEI Candidate Genes without Filter.

**Additional file 2: Table S2.** IEI Candidate Genes with high (>.9) pLI Filter.

**Additional file 3: Table S3.** IEI Candidate Genes with Average in all Immune Cells > 0 TPM Filter.

**Additional file 4: Table S4.** IEI Candidate Genes with Average in B Cells > 1 TPM Filter.

**Additional file 5: Table S5.** IEI Candidate Genes with Average in DCs > 1 TPM Filter.

**Additional file 6: Table S6.** IEI Candidate Genes with Average in Myeloid Cells > 1 TPM Filter.

**Additional file 7: Table S7.** IEI Candidate Genes with Average in NK Cells > 1 TPM Filter.

**Additional file 8: Table S8.** IEI Candidate Genes with Average in T Cells > 1 TPM Filter.
