# Supplementary material for: Expanding the potential genes of inborn errors of immunity through protein interactions
Source: BMC Genomics. 2021 Aug 15;22:618. doi: 10.1186/s12864-021-07909-3 (PMC8364696; doi:10.1186/s12864-021-07909-3)
Supplement: Supplementary file 9 — Additional file 9: Figure S1. Algorithm verification using out-of-sample testing. (A) Percentage of validation set genes rediscovered by algorithm. (B) Percentage of genes from left-out category rediscovered by algorithm. Figure S2. Post-translational modifications induced by and received by IEI gene products. (A) The number of targets or substrates for each IEI gene when they are either a kinase, phosphatase, protease, or ubiquitinase. (B) The number of proteins that are known to target IEI proteins for post-translational modification by various mechanisms. Figure S3. Human Gene Connectome (HGC) Distance between known and candidate IEI genes. Figure S4. Known IEI-causative genes have varying levels of predicted mutational harm. (A) Confirmed IEI gene pLI disaggregated by inheritance type (AD: autosomal dominant, AR: autosomal recessive). (B) Confirmed IEI gene Gene Damage Indices (GDIs) disaggregated by inheritance type. (C) Damage prediction of confirmed IEI genes as classified by the GDI Server. (D) Known IEI gene pLIs plotted against GDIs and disaggregated by inheritance type. Figure S5. Known IEI transcripts skew to higher expression in immune cell types. (A) RNAseq expression of all Human Blood Atlas-recorded genes disaggregated by cell types present. (B) RNAseq expression of all IEI genes disaggregated by cell type. Line drawn at 1 TPM (at 2 when transformed to TPM+1). Percentage above 1 TPM (at 2 when transformed to TPM+1) cutoff presented. [file 12864_2021_7909_MOESM9_ESM.pdf]

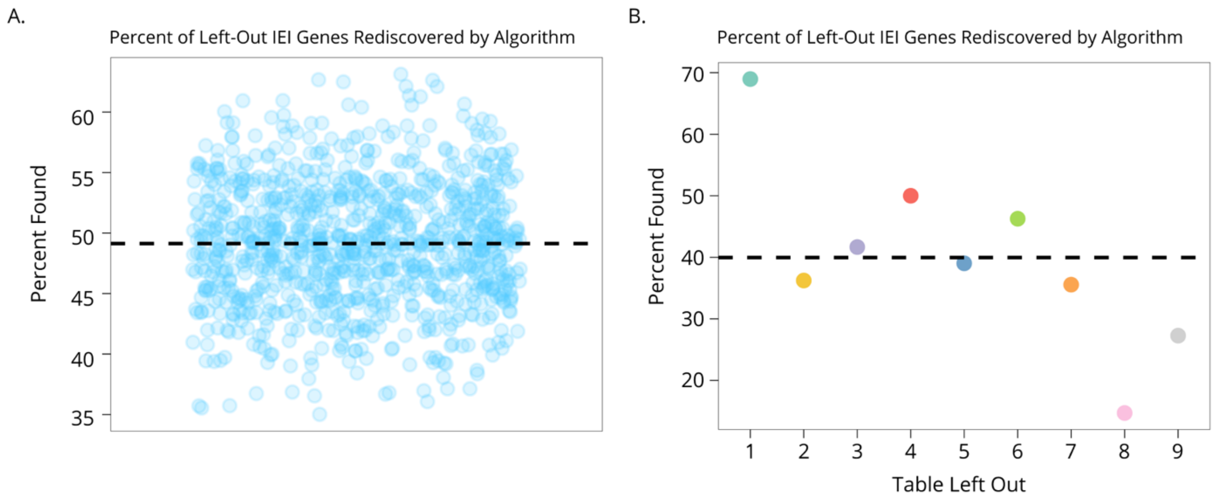

Figure S1. Algorithm verification using out-of-sample testing. (A) Percentage of validation set genes rediscovered by algorithm. **(B)** Percentage of genes from left-out category rediscovered by algorithm.

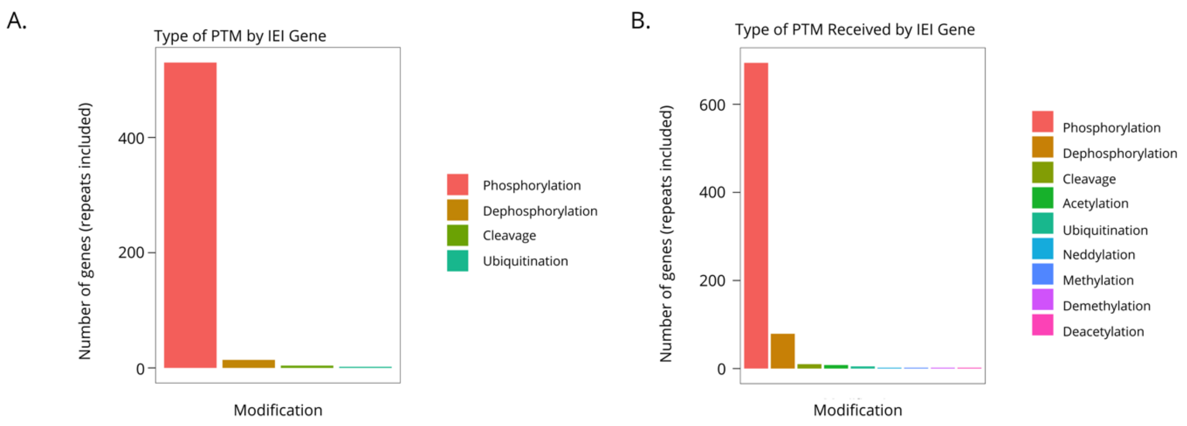

Figure S2. Post-translational modifications induced by and received by IEI gene products. **(A)** The number of targets or substrates for each IEI gene when they are either a kinase, phosphatase, protease, or ubiquitinase. **(B)** The number of proteins that are known to target IEI proteins for post-translational modification by various mechanisms.

A.

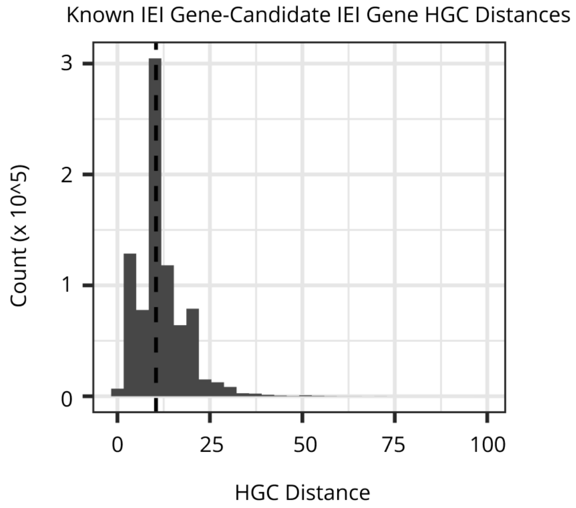

Figure S3. Human Gene Connectome (HGC) Distance between known and candidate IEI genes.

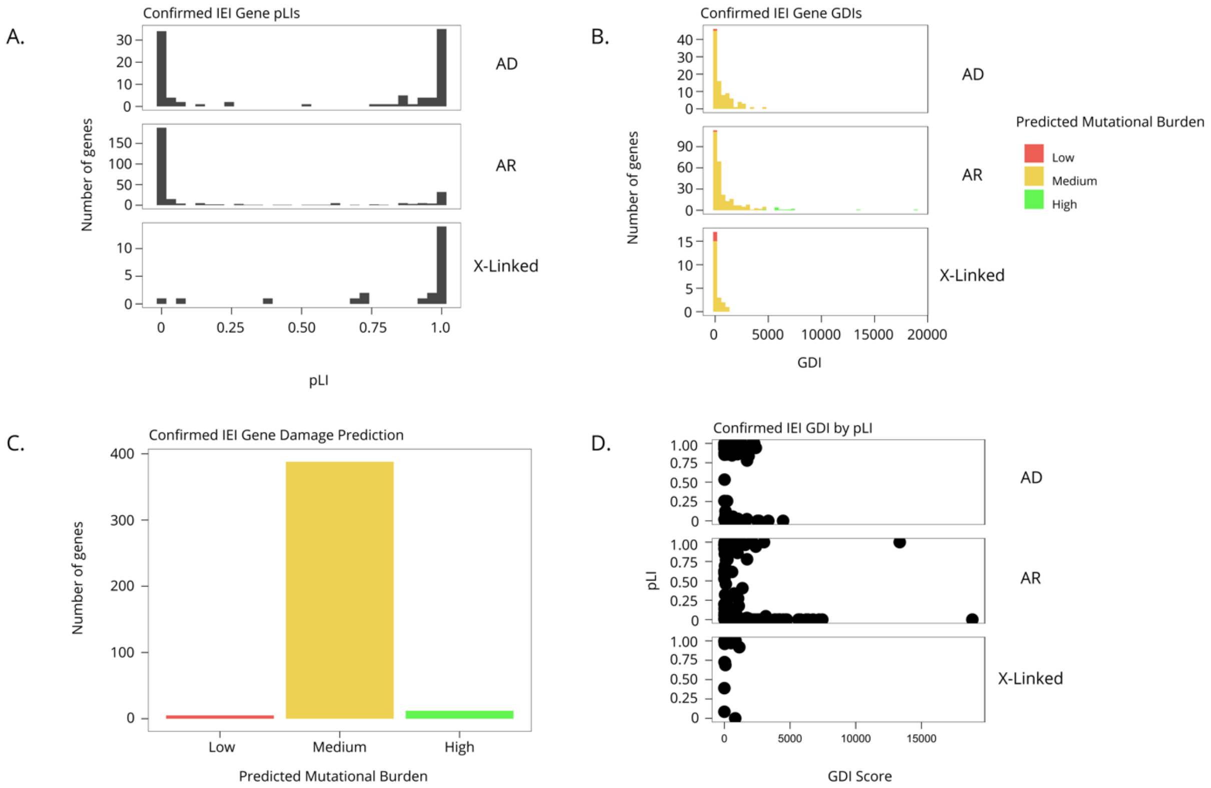

Figure S4. Known IEI-causative genes have varying levels of predicted mutational harm. (A) Confirmed IEI gene pLI disaggregated by inheritance type (AD: autosomal dominant, AR: autosomal recessive). **(B)** Confirmed IEI gene Gene Damage Indices (GDIs) disaggregated by inheritance type. **(C)** Damage prediction of confirmed IEI genes as classified by the GDI Server. **(D)** Known IEI gene pLIs plotted against GDIs and disaggregated by inheritance type.

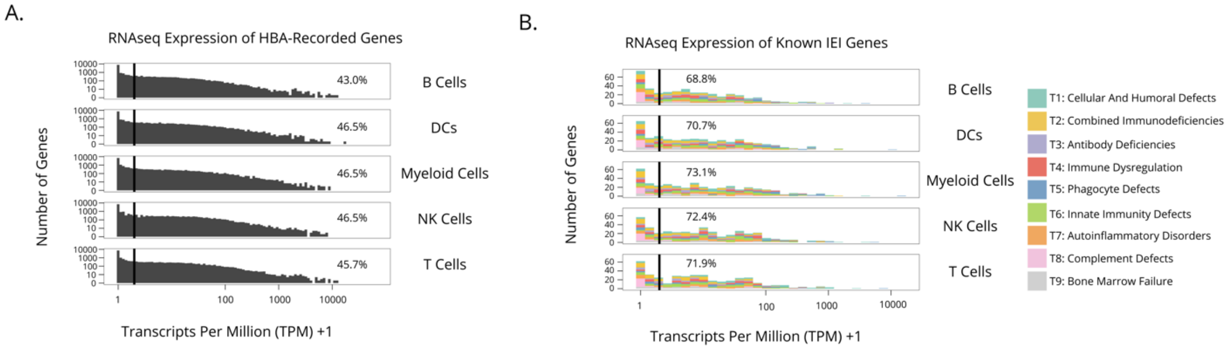

Figure S5. Known IEI transcripts skew to higher expression in immune cell types. (A) RNAseq expression of all Human Blood Atlas-recorded genes disaggregated by cell types present. **(B)** RNAseq expression of all IEI genes disaggregated by cell type. Line drawn at 1 TPM (at 2 when transformed to TPM+1). Percentage above 1 TPM (at 2 when transformed to TPM+1) cutoff presented.
